# Age-dependent interactions of APOE isoform 4 and Alzheimer’s disease neuropathology: findings from the NACC

**DOI:** 10.1186/s40478-025-02012-0

**Published:** 2025-05-17

**Authors:** Cellas A. Hayes, Roland J. Thorpe, Michelle C. Odden

**Affiliations:** 1 School of Medicine Department of Epidemiology and Population Health, Stanford University, 1701 Page Mill Road, Palo Alto, CA 94304 USA; 2 Program for Research on Men’s Health, Johns Hopkins Center for Health Disparities Solutions, Johns Hopkins Bloomberg School of Public Health, 624 N. Broadway, Suite 441, Baltimore, MD 21205 USA; 3 Johns Hopkins Alzheimer’s Disease Resource Center for Minority Aging Research, Johns Hopkins Bloomberg School of Public Health, 624 N. Broadway, Room 388, Baltimore, MD 21205 USA

**Keywords:** Alzheimer’s disease related pathology, Lewy body disease, Hippocampal sclerosis, Neuropathology, Arteriolosclerosis, Infarcts

## Abstract

Alzheimer’s disease related pathologies, neurodegenerative pathologies, and vascular neuropathologies are common in older adults at death. Previous studies using the National Alzheimer’s Coordinating Center (NACC) have not investigated the association between age at death and apolipoprotein E (*APOE*) ε4 and the prevalence of neuropathologies found at autopsy. We used autopsy confirmed neuropathology data from the NACC to examine the interactive effects of age and *APOE* ε4 on various neuropathologies (N = 5,843) using modified Poisson regression to estimate the prevalence ratios. Significant interactions between *APOE* ε4 and age at death were observed for neuritic plaques, Braak staging, diffuse neuritic plaques, and Lewy body disease pathology, with the effect of *APOE* ε4 decreasing at older ages. In contrast, a significant positive interaction was found for hemorrhages/microbleeds, indicating that the association between *APOE* ε4 and microbleeds strengthens with increasing age. These findings suggest that future therapeutic strategies should consider both genetic risk and age to effectively target AD progression.

**Supplementary Information:**

The online version contains supplementary material available at 10.1186/s40478-025-02012-0.

## Introduction

Beyond age, the apolipoprotein E (*APOE*) gene is the greatest risk determinant of late-onset Alzheimer’s disease (LOAD) in which symptoms develop after the age of 65 [1]. Previous studies have shown that the *APOE* ε2 isoform is associated with a decreased risk of developing Alzheimer’s disease (AD) dementia [2, 3]. Conversely, the *APOE* ε4 isoform carries the greatest risk in the development of LOAD [4]. The *APOE* gene has a dose-dependent relationship with AD. Specifically, individuals who are *APOE* ε4 positive have an earlier age of onset of AD compared to the ε3 and ε2 isoforms [4, 5].

Generally, *APOE* functions as a lipid binding protein and is the primary cholesterol transporter [6]. Yet, there is substantial evidence showing that *APOE* is a central driver of AD pathology accumulation—amyloid-β plaques and neurofibrillary tangles [7]. Studies have also shown that *APOE* can serve as a risk factor for other neurodegenerative diseases. For example, *APOE* was previously associated with an increased risk of dementia with Lewy bodies (DLB) and Parkinson’s disease [8–13]. Additionally, *APOE* has been associated with transactive response DNA-binding protein 43 (TDP-43) pathology in Alzheimer’s disease brains [14–16]. These findings underscore the significant role of *APOE* not only in AD but also in a broader spectrum of neurodegenerative disorders, highlighting its relevance as a key genetic risk factor across multiple neuropathologies.

The National Alzheimer’s Coordinating Center (NACC) serves as a central repository for data collected from the Alzheimer’s disease research centers (ADRC) in the United States. In individuals with AD, the *APOE* ε4 allele is over-represented with over 60% of individuals that are diagnosed with AD being ε4 positive [3, 5, 7, 17]. Therefore, there is a large percentage of ADRC participants that carry at least one *APOE* ε4 allele [18]. In addition, the NACC neuropathology dataset is the largest repository of autopsy-derived data relevant to neurodegenerative diseases [19]. Thus, it provides a unique opportunity to investigate the association between *APOE* ε4 and confirmed neuropathology cases on a more accurate scale than in vivo studies.

Previous NACC findings demonstrated that the prevalence of pure AD and Lewy body disease (LBD) pathology decreases with age, whereas microinfarcts, TDP-43, hippocampal sclerosis, gross infarcts, and microhemorrhages increase with age in individuals with AD neuropathologic change (intermediate or high AD neuropathological change level (ADNC)) [20]. However, this study used logistic regression to estimate the associations, which overestimates the relative risk when the outcome is common as in the NACC registry [21, 22]. In this study, we employed modified Poisson regression that more accurately estimates relative risk, particularly when the outcome is common, to account for the high prevalence of pathologies in the sample and to obtain precise prevalence rate ratios. Although there are strong associations between mixed pathology/polypathology and age, there remains limited evidence evaluating an interaction between age and *APOE* ε4 on neuropathology beyond AD. To build on the work of Beach and colleagues, our objective was to investigate how age and *APOE* ε4 interact to influence the prevalence of major neuropathologies, including neuritic plaques, neurofibrillary tau, diffuse plaques, LBD, TDP-43, hippocampal sclerosis, and vascular neuropathologies (arteriolosclerosis, atherosclerosis of the circle of Willis, cerebral amyloid angiopathy (CAA), infarcts/lacunes, microinfarcts, and hemorrhages/microbleeds). This study is a strong comprehensive evaluation of the interaction between age and *APOE* ε4 on autopsied-confirmed neuropathologies in a large clinical cohort including vascular neuropathologies irrespective of clinical diagnosis.

## Materials and methods

### NACC and research participants selection

The NACC serves as a repository for participant information gathered from Alzheimer’s Disease Research Centers (ADRC), which are funded by the National Institute on Aging. The ADRC enrolls participants at all cognitive stages (normal, impaired not mild cognitively impairment, mild cognitive impairment (MCI), and dementia) and does not have a specific neurodegenerative disease focus. However, the majority of participants are diagnosed with AD and are enrolled when cognitive decline has begun.

Each individual ADRC receives independent institutional review board approval for their respective studies. We analyzed data from the March 2023 data freeze for the NACC Uniform Data Set (UDS). Following the exclusion criteria, 5,843 participants were eligible for this analysis and were from 37 ADRC. In this study, our sample focused specifically on participants who had undergone autopsies to quantify neuropathology. Table 1 contains the summary of the NACC-specific neuropathology variables, their descriptions, and the dichotomization method. For neuropathology variables that were not coded as dichotomous, we modified these variables to indicate the absence (none) versus the presence (any level) of a specific pathology found at autopsy. The original documentation for all NACC variables can be found online through the NACC documentation pages, including details on all variables and the autopsy neuropathology dataset. The NACC Handbook: A Researcher’s Guide is available at: https://naccdata.org/requesting-data/data-request-process.

**Table 1:** Definitions and dichotomous criteria for neuropathological variables

Variable Name	Definition	NACC Neuropathology v.10 Variable	Dichotomous Criteria	
			0	1
Neuritic plaques	Density of neocortical neuritic plaques (CERAD score) (C score)	NACCNEUR	Absent	Sparse/Inter./Freq
Braak staging	Braak stage for neurofibrillary degeneration (B score*)*	NACCBRAA	Absent	B1/B2/B3
Diffuse plaques	Density of diffuse plaques (CERAD semiquantitative score)	NACCDIFF	Absent	Sparse/Inter./Freq
LBD pathology	Lewy body pathology derived	NACCLEWY	Absent	Brainstem predominant/limbic (transitional) or amygdala-predominant/neocortical (diffuse)
TDP-43	pTDP-43 in amygdala, hippocampus, entorhinal/inferior temporal cortex, or neocortex	NPTDPB, NPTDPC, NPTDPD, NPTDPE	Absent	Present (any region)
Hippocampal sclerosis	Hippocampal sclerosis (CA1 and/or subiculum)	NPHIPSCL	Absent	Present
Arteriolosclerosis	Arteriolosclerosis	NACCARTE	Absent	Mild/Moderate/Severe
Atherosclerosis of the circle of Willis	Atherosclerosis of the circle of Willis	NACCAVAS	Absent	Mild/Moderate/Severe
CAA	Cerebral amyloid angiopathy	NACCAMY	Absent	Mild/Moderate/Severe
Infarcts/lacunes	Infarcts and lacunes	NACCINF	Absent	Present
Microinfarcts	Microinfarcts	NACCMICR	Absent	Present
Hemorrhages/microbleeds	Hemorrhages and microbleeds	NACCHEM	Absent	Present

NACC National Alzheimer’s Coordinating Center; CERAD Consortium to Establish a Registry for Alzheimer’s Disease; Braak Staging method to classify the severity of neurofibrillary tangles in Alzheimer’s disease; ADNC Alzheimer’s Disease Neuropathologic Change; NIA-AA National Institute on Aging–Alzheimer’s Association; LBD Lewy Body Disease; TDP-43 Transactive Response DNA-binding Protein of 43 kDa; CAA Cerebral Amyloid Angiopathy; CA1 Cornu Ammonis 1 (a region of the hippocampus); inter. Intermediate; Freq. frequent; Mod. Moderate; Sev. Severe

This table outlines key neuropathological variables and their definitions as per the National Alzheimer’s Coordinating Center (NACC) Neuropathology v.10 dataset. Each variable is paired with its corresponding NACC code and categorized into dichotomous criteria. “0” indicates the absence or low levels of pathology, while “1” represents the presence or high levels of pathology, based on standardized scoring systems such as CERAD, Braak staging, and NIA-AA guidelines. This classification aids in evaluating the presence and severity of neuropathological changes associated with Alzheimer’s disease and related conditions

### Exclusion criteria

The data received from the NACC included 47,772 participants, and the autopsied sample was 7,476. First, 856 participants with missing values for *APOE* ε4 were removed, resulting in 6,620 participants. Subsequently, 732 participants with *APOE* alleles of ε2ε2 or ε2ε3 or ε2ε4 were removed, leaving a sample of 5,888 participants. The justification for excluding participants with the ε2 allele is its protective effects against AD pathology accumulation [23, 24]. The last exclusion criterion was the removal of 45 participants who were under the age of 50 at death due to the small sample size, resulting in 5,843 participants.

### Pre-mortem clinical etiological diagnosis

In this study of 5,843 autopsied ADRC participants, the pre-mortem clinical etiological diagnoses among the participants are described in Supplemental Table 1. An AD diagnosis was the most prevalent diagnosis across all decadal groups, increasing from 42.1% in the 50–59 age group to 67.3% in the 80–89 age group, before slightly declining to 63.4% in the 90 + group, resulting in an overall prevalence of 59.2%. LBD was the second most common diagnosis, with an overall prevalence of 6.9%, peaking in the 70–79 group (11.1%). Frontotemporal lobar degeneration, including motor neuron disease, had an overall prevalence of 10.9%. Other diagnoses such as vascular brain injury and corticobasal degeneration had relatively low prevalence rates, with overall rates of 2.6% and 1.7%, respectively. The percentage of participants categorized as “Not Cognitively Impaired” increased with age, reaching 21.7% in the 90 + group, contributing to an overall rate of 11.6%. Rare conditions like Down Syndrome, Huntington’s Disease, and central nervous system neoplasms were nearly absent, with rates below 0.1%.

### Age at death groups

The sample was divided into age at death decadal groups following previous methodology in another NACC neuropathology investigation [25]. Our sample consisted of 5,843 participants, stratified into five decadal age groups: 50–59 years (N = 202), 60–69 years (N = 842), 70–79 years (N = 1,399), 80–89 years (N = 2,043), and 90 + years (N = 1,357). For statistical purposes, age at death was treated as a continuous variable. The age at death decadal bins were used to showcase clinical etiological diagnoses and sample characteristics.

### APOE

Participants with the ε3ε3 genotype were coded as 0, indicating the absence of an *APOE* ε4 allele. Participants who were heterozygous and homozygous for ε4 were coded as 1, indicating the presence of the *APOE* ε4 allele. Across the sample, 46.2% of participants carried at least one *APOE* ε4 allele. The proportion of ε4 carriers varied across age groups: 43.6% in the 50–59 age group, 47.1% in the 60–69 age group, 55.2% in the 70–79 age group, 49.9% in the 80–89 age group, and 31.2% in the 90 + age group. The ε3/ε3 genotype was the most common overall, accounting for 53.8% of the sample. Its prevalence ranged from 44.8% in the 70–79 age group to 68.8% in the 90 + age group. The ε3/ε4 genotype was observed in 37.0% of participants, with the highest prevalence in the 70–79 (40.7%) and 80–89 (41.1%) age groups, and the lowest in the 90 + age group (28.4%). The ε4/ε4 genotype was found in 9.2% of the overall sample, with the highest prevalence in the 70–79 age group (14.5%) and lowest in the 90 + age group (2.9%).

### Model covariates

For the statistical analyses, we controlled for sex (female/male) and the number of years of education (centered). In the sample, 94.4% were White and 3.5% identified as Hispanic. Thus, we did not control for race or ethnicity in the statistical models.

### Neuropathology characterization and dichotomization

The ADRC conducted neuropathology assessments during autopsies using consensus guidelines with neuropathology forms 9 and 10. Currently, form 11 is used for these assessments, and the data are subsequently uploaded to the NACC registry for distribution [26, 27]. To align with our objective, we dichotomized the neuropathological assessments. Previous studies have used dichotomization of neuropathology as a predictor and outcome in NACC since low levels of pathology are not considered to contribute significantly to cognitive or functional deficits [20, 25, 28–32]. However, to further align with our objective and focus on neuropathology prevalence, we used a dichotomous variable of none versus any pathology. Table 1 summarizes the neuropathological variables examined in this study, with each variable dichotomized to indicate either the presence or absence, or categorized as absent versus present (sparse, intermediate, or frequent), or absent versus present (mild, moderate, or severe), based on the original pathology classification from NACC.

Neuropathologic evaluation of AD-related pathologies was conducted according to standardized protocols [27]. The density of neocortical neuritic plaques, measured by the CERAD score (NACCNEUR), was categorized as absent or present (sparse, intermediate or frequent). Neurofibrillary tangles for tau (NACCBRAA) were categorized using Braak staging and was originally coded as stage 0 (B0), stage I/II (B1), stage III/IV (B2), and stage V/VI (B3) [33]. Braak staging for neurofibrillary tangles (NACCBRAA) was dichotomized into absent or present (B1, B2, or B3). Diffuse plaques (CERAD) semiquantitative score (NACCDIFF) was classified as none versus any (sparse, intermediate or frequent) [19].

LBD pathology (NACCLEWY) was classified as absent or present (brainstem-predominant, limbic, amygdala-predominant, or neocortical) [34]. Additionally, the presence of phosphorylated TDP-43, assessed across the amygdala, hippocampus, entorhinal/inferior temporal cortex, and neocortex (NACC variables: NPTDPB, NPTDPC, NPTDPD, NPTDPE), was dichotomized as absent or present [35]. Hippocampal sclerosis (NPHIPSCL) was categorized as absent or present [36].

Vascular neuropathologies included in this study include arteriolosclerosis, atherosclerosis of the circle of Willis, cerebral amyloid angiopathy (CAA), infarcts/lacunes, microinfarcts, and hemorrhages/microbleeds. We utilized the derived variables across all the ADRC for vascular neuropathologies to increase reproducibility. Arteriolosclerosis (NACCARTE) [37] and atherosclerosis (NACCAVAS) [38] were classified as absent versus present (mild, moderate or severe). CAA (NACCAMY) followed similar criteria [39]. Additional vascular-related pathologies, including infarcts and lacunes (NACCINF) [40] [41], microinfarcts (NACCMICR) [42], and hemorrhages/microbleeds (NACCHEM) [43], were all originally collected as either absent or present.

### Statistical analyses

Demographic and neuropathology characteristics of the sample were analyzed to assess differences between age groups using a one-way ANOVA/Tukey post hoc test or chi-square test for continuous variables and categorical variables, respectively.

### Modified poisson regressions

The dependent variable in our models was the presence or absence of specific neuropathologies. Because many of these outcomes have a prevalence that approaches 50%, we used modified Poisson regression models [21, 22, 44]. This method was chosen because traditional logistic regression overestimates the relative risk when the outcome is common [21, 22]. In each model, we included educational attainment, sex, *APOE* ε4 status, age at death, and an interaction between *APOE* ε4 status and age at death. Following significant interactions, we performed stratified analyses by *APOE* ε4 status.

To further investigate sex differences, we ran separate modified Poisson regression models including a three-way interaction term between *APOE* ε4 status, age at death, and sex. Prevalence rate ratios (PRR) and 95% confidence intervals (CI) were calculated for each coefficient, facilitating interpretation of the interaction between *APOE* ε4 and age at death. This approach allows for a more accurate estimation of relative risk in the context of prevalent binary outcomes.

### Sensitivity analysis for birth cohort effects

To assess the potential impact of birth cohort effects on our findings, we conducted a sensitivity analysis by incorporating birth cohort as a categorical variable in our models. Birth years were categorized into seven birth cohorts using predefined breakpoints: ≤ 1909 (n = 33), 1910–1919 (n = 605), 1920–1929 (n = 1896), 1930–1939 (n = 1593), 1940–1949 (n = 1133), 1950–1959 (n = 512), and 1960 + (n = 71). The oldest cohort (≤ 1909) was selected as the reference category to facilitate comparisons across age groups. The birth cohort variable was included as a covariate in the modified Poisson regression models evaluating neuropathological outcomes, alongside key predictors including years of education (centered), sex, and the interaction term between *APOE* ε4 and age at death.

All statistical analyses were conducted using R version 4.2.3. All p-values were two-sided, and the statistical significance was set at *p*-value < 0.05 for all comparisons.

## Results

### Sample description

The study included 5,843 participants spanning five age groups at death (50–59, 60–69, 70–79, 80–89, and 90 + years) (Table 2). The mean age at death was 80.7 years (SD = 10.9), with participants having an average of 15.4 years of education (SD = 3.1). The cohort was predominantly White (94.4%), with 53.2% male participants. *APOE* ε4 carriers comprised 46.2% of the total sample, with the highest prevalence observed in the 70–79 age group (55.2%). Regarding neuropathological findings, 52.7% of participants had high Braak stage (B3), 46.9% had frequent amyloid pathology, and 64.2% showed no LBD pathology. Vascular pathologies were also common, with 77.0% having atherosclerosis and arteriolosclerosis (40.8%), overall. Cognitive status prior to death indicated that 78.6% of participants had dementia, while 11.6% remained cognitively normal**.**

**Table 2:** Descriptive characteristics and prevalence of neuropathologies across decadal age groups at death

	50–59 Years (N = 202)	60–69 Years (N = 842)	70–79 Years (N = 1399)	80–89 Years (N = 2043)	90 + Years (N = 1357)		Overall (N = 5843)
*APOE* ε4, n (%)	88 (43.6)	397 (47.1)	772 (55.2)	1020 (49.9)	424 (31.2)	< 0.001	2701 (46.2)
*APOE Haplotype*, n (%)						< 0.001	
ε3/ε3	114 (56.4)	445 (52.9)	627 (44.8)	1023 (50.1)	933 (68.8)		3142 (53.8)
ε3/ε4	76 (37.6)	293 (34.8)	569 (40.7)	840 (41.1)	385 (28.4)		2163 (37.0)
ε4/ε4	12 (5.9)	104 (12.4)	203 (14.5)	180 (8.8)	39 (2.9)		538 (9.2)
*Demographic Characteristics*							
Age at Death (years) Mean (SD)	56.0 (2.5)	65.1 (2.8)	74.9 (2.8)	84.7 (2.8)	94.0 (3.6)	< 0.001	80.7 (10.9)
Years of Education Mean (SD)	15.0 (2.7)	15.5 (2.9)	15.6 (3.0)	15.5 (3.2)	15.2 (3.3)	0.01	15.4 (3.1)
Missing, n (%)	6 (3.0)	13 (1.5)	18 (1.3)	11 (0.5)	12 (0.9)		60 (1.0)
Race, n (%)						0.01	
White	193 (95.5)	793 (94.2)	1331 (95.1)	1927 (94.3)	1273 (93.8)		5517 (94.4)
Black	5 (2.5)	25 (3.0)	43 (3.1)	92 (4.5)	61 (4.5)		226 (3.9)
American Indian or Alaskan Native	0 (0)	1 (0.1)	2 (0.1)	3 (0.1)	2 (0.1)		8 (0.1)
Native Hawaiian or other Pacific Islander	0 (0)	1 (0.1)	4 (0.3)	1 (0.0)	0 (0)		6 (0.1)
Asian	3 (1.5)	10 (1.2)	5 (0.4)	13 (0.6)	13 (1.0)		44 (0.8)
Other	0 (0)	3 (0.4)	6 (0.4)	4 (0.2)	6 (0.4)		19 (0.3)
Unknown	1 (0.5)	9 (1.1)	8 (0.6)	3 (0.1)	2 (0.1)		23 (0.4)
Hispanic Ethnicity, n (%)	7 (3.5)	28 (3.3)	45 (3.2)	78 (3.8)	46 (3.4)	0.89	204 (3.5)
Unknown Hispanic, n (%)	1 (0.5)	7 (0.8)	6 (0.4)	8 (0.4)	7 (0.5)		29 (0.5)
Male, n (%)	119 (58.9)	506 (60.1)	825 (59.0)	1108 (54.2)	551 (40.6)	< 0.001	3109 (53.2)
*AD—Related Neuropathologies, n (%)*							
Neuritic plaques (Sparse/Intermediate/High)	113 (55.9)	602 (71.5)	1110 (79.3)	1721 (84.2)	1061 (78.2)	< 0.001	4607 (78.8)
Missing	3 (1.5)	5 (0.6)	7 (0.5)	5 (0.2)	0 (0)		20 (0.3)
Braak staging (B1/B2/B3)	140 (69.3)	718 (85.3)	1282 (91.6)	1994 (97.6)	1320 (97.3)	< 0.001	5454 (93.3)
Missing	11 (5.4)	22 (2.6)	36 (2.6)	20 (1.0)	16 (1.2)		105 (1.8)
Diffuse plaques (Sparse/Intermediate/High)	124 (61.4)	635 (75.4)	1127 (80.6)	1677 (82.1)	1067 (78.6)	< 0.001	4630 (79.2)
Missing	11 (5.4)	51 (6.1)	105 (7.5)	189 (9.3)	131 (9.7)		487 (8.3)
*Neurodegenerative Neuropathologies, n (%)*							
LBD pathology (any)	42 (20.8)	262 (31.1)	519 (37.1)	691 (33.8)	318 (23.4)	< 0.001	1832 (31.4)
Missing	13 (6.4)	44 (5.2)	63 (4.5)	101 (4.9)	41 (3.0)		262 (4.5)
TDP-43 (any)	22 (10.9)	120 (14.3)	245 (17.5)	338 (16.5)	238 (17.5)	0.001	963 (16.5)
Missing	138 (68.3)	531 (63.1)	851 (60.8)	1358 (66.5)	896 (66.0)		3774 (64.6)
Hippocampal sclerosis	4 (2.0)	48 (5.7)	99 (7.1)	195 (9.5)	127 (9.4)	< 0.001	473 (8.1)
Missing	106 (52.5)	364 (43.2)	609 (43.5)	955 (46.7)	567 (41.8)		2601 (44.5)
*Vascular Neuropathologies, n (%)*							
Arteriolosclerosis (Mild/Moderate/Severe)	39 (19.3)	255 (30.3)	514 (36.7)	914 (44.7)	662 (48.8)	< 0.001	2384 (40.8)
Missing	13 (6.4)	75 (8.9)	139 (9.9)	230 (11.3)	131 (9.7)		588 (10.1)
Atherosclerosis of the circle of Willis (Mild/Moderate/Severe)	77 (38.1)	473 (56.2)	1032 (73.8)	1701 (83.3)	1216 (89.6)	< 0.001	4499 (77.0)
Missing	7 (3.5)	16 (1.9)	24 (1.7)	26 (1.3)	8 (0.6)		81 (1.4)
Cerebral amyloid angiopathy (Mild/Moderate/Severe)	86 (42.6)	481 (57.1)	865 (61.8)	1316 (64.4)	787 (58.0)	< 0.001	3535 (60.5)
Missing	7 (3.5)	15 (1.8)	37 (2.6)	34 (1.7)	21 (1.5)		114 (2.0)
Infarcts/lacunes (present)	7 (3.5)	62 (7.4)	159 (11.4)	461 (22.6)	351 (25.9)	< 0.001	1040 (17.8)
Missing	5 (2.5)	15 (1.8)	16 (1.1)	12 (0.6)	3 (0.2)		51 (0.9)
Microinfarcts (present)	7 (3.5)	87 (10.3)	208 (14.9)	465 (22.8)	421 (31.0)	< 0.001	1188 (20.3)
Missing	6 (3.0)	16 (1.9)	17 (1.2)	8 (0.4)	2 (0.1)		49 (0.8)
Hemorrhages/ Microbleeds (present)	2 (1.0)	42 (5.0)	79 (5.6)	134 (6.6)	75 (5.5)	0.03	332 (5.7)
Missing	14 (6.9)	56 (6.7)	50 (3.6)	37 (1.8)	10 (0.7)		167 (2.9)
*Cognitive Status Proximate to Death, n (%)*						< 0.001	
Normal	9 (4.5)	21 (2.5)	100 (7.1)	255 (12.5)	295 (21.7)		680 (11.6)
Impaired not MCI	0 (0)	8 (1.0)	12 (0.9)	35 (1.7)	30 (2.2)		85 (1.5)
MCI	7 (3.5)	28 (3.3)	51 (3.6)	178 (8.7)	220 (16.2)		484 (8.3)
Dementia	186 (92.1)	785 (93.2)	1236 (88.3)	1575 (77.1)	812 (59.8)		4594 (78.6)

APOE apolipoprotein E; SD standard deviation; LBD Lewy Body Disease; TDP-43 Transactive Response DNA-binding Protein of 43 kDa; MCI mild cognitive impairment

Data were statistically compared across age bins using one-way ANOVA/Tukey posthoc for continuous variables or chi-square test for categorial variables

### Interactions between APOE ε4 and age at death on neuropathological markers

**Fig. 1:**
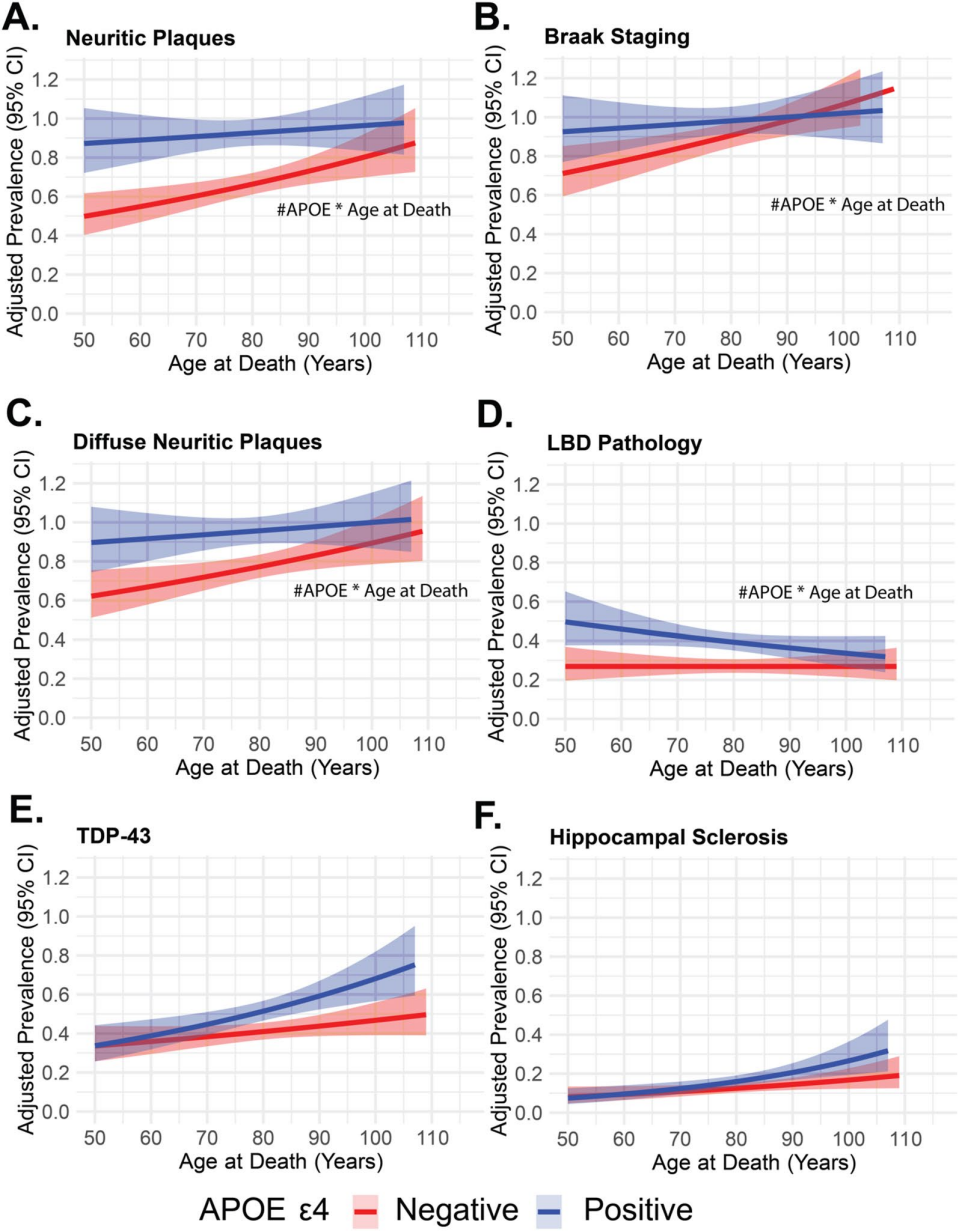
Adjusted prevalence of neuropathological changes across age at death stratified by APOE ε4 status. **A**–**F **illustrate the adjusted prevalence of various Alzheimer’s disease related neuropathological markers and other major neurodegenerative pathologies across age at death. Red lines indicate those without the APOE ε4 allele (*APOE* ε4 negative) while blue lines represent individuals with *APOE* ε4 positivity. The adjusted prevalence was derived from modified Poisson regression models adjusted for education level and sex and included the interaction term between APOE ε4 and age at death, with robust standard errors applied to account for heteroscedasticity. The line represents the mean adjusted prevalence, and the surrounding band denote 95% confidence intervals calculated from robust standard errors. **A**: Prevalence of neuritic plaques based on the CERAD score (none versus sparse, intermediate, and frequent). **B**: Prevalence of Braak staging (none versus low (B1), intermediate (B2), and high (B3)). **C**: Prevalence of diffuse neuritic plaques based on the CERAD semiquantitative score (none, sparse, intermediate, and frequent). **D**: Prevalence of Lewy body disease (LBD) pathology (none versus brainstem predominant, limbic or amygdala-predominant, and neocortical). **E**: Prevalence of TDP-43 proteinopathy in any region. **E**: Prevalence of hippocampal sclerosis presence. The hash symbol (#APOE x Age at Death) indicates significant interactions between *APOE* ε4 positivity and age at death. Abbreviations: CI confidence interval; APOE ε4 Apolipoprotein E epsilon 4 allele; CERAD Consortium to Establish a Registry for Alzheimer’s Disease; Braak staging classification of neurofibrillary tangle severity; LBD Lewy body disease; TDP-43 transactive response DNA binding protein 43

**Fig. 2:**
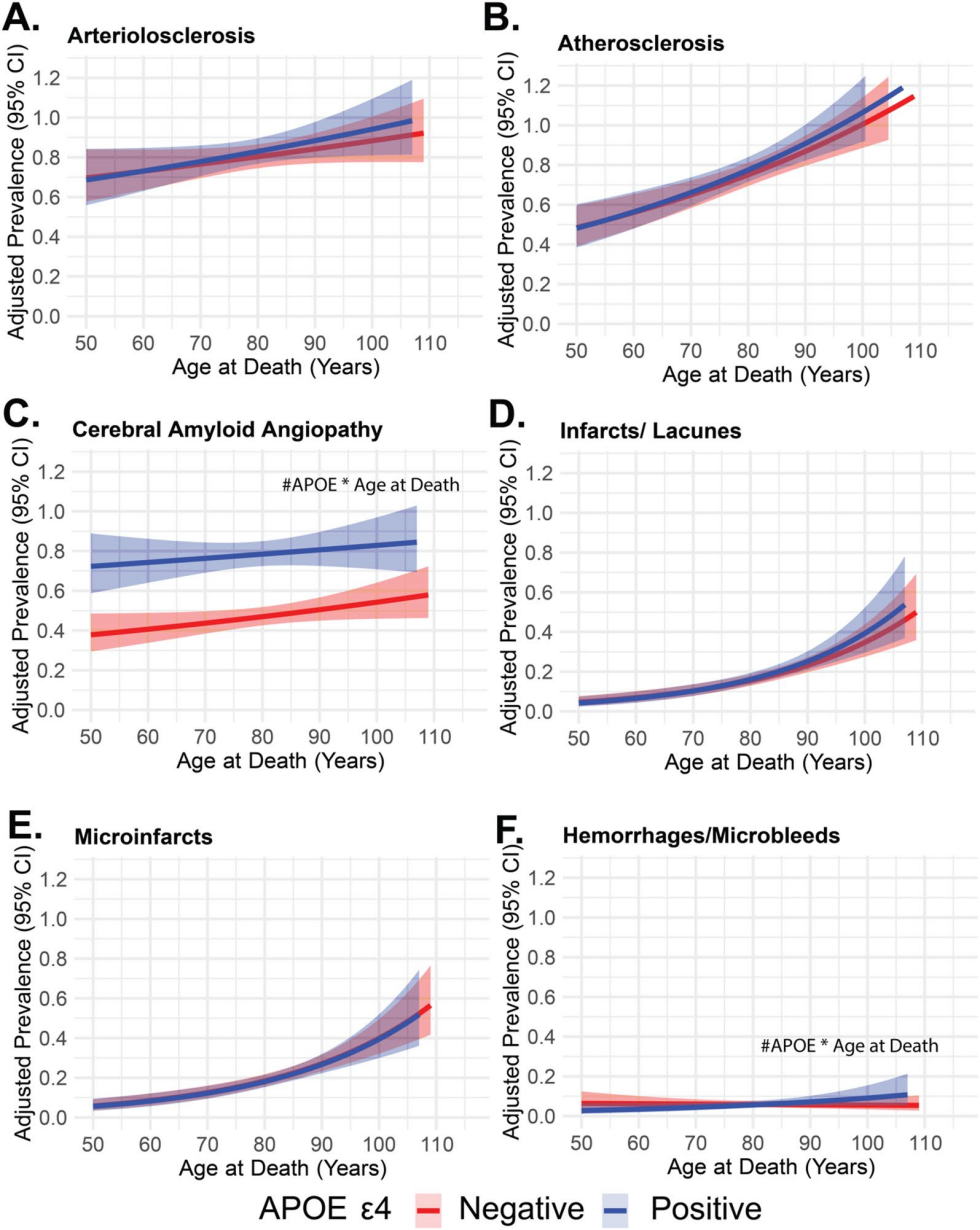
Adjusted prevalence of vascular neuropathologies across age groups stratified by APOE ε4 status. **A**–**F** display the adjusted prevalence of vascular and cerebrovascular pathologies across age at death. Red lines indicate those without the APOE ε4 allele (*APOE* ε4 negative) while blue lines represent individuals with *APOE* ε4 positivity. The adjusted prevalence was derived from modified Poisson regression models adjusted for education level and sex and included the interaction term between APOE ε4 and age at death, with robust standard errors applied to account for heteroscedasticity. The line represents the mean adjusted prevalence, and the surrounding band denote 95% confidence intervals calculated from robust standard errors. **A**: Prevalence of arteriolosclerosis (none versus mild, moderate, and severe). **B**: Prevalence of atherosclerosis of the circle of Willis (none versus mild, moderate, and severe). **C**: Prevalence of cerebral amyloid angiopathy (CAA) (none versus mild, moderate, and severe). **D**: Prevalence of the presence of infarcts or lacunes. **E**: Prevalence of the presence of microinfarcts. **F**: Prevalence of the presence of hemorrhages/microbleeds. The hash symbol (#APOE x Age at Death) indicates significant interactions between *APOE* ε4 positivity and age at death. Abbreviations: CI confidence interval; APOE ε4 Apolipoprotein E epsilon 4 allele; Atherosclerosis Atherosclerosis of the circle of Willis

In our analysis examining the interaction between *APOE ε4* status and age at death**,** we observed significant findings across multiple neuropathological outcomes (Fig. 1, Supplemental Table 3). Notably, significant negative interactions between *APOE ε4* and age at death were observed for neuritic plaques (PRR: 0.99, 95% CI 0.99–1.00, *p* < 0.001), Braak stage (PRR: 0.99, 95% CI 0.99–1.00, *p* < 0.001), diffuse plaques (PRR: 0.99, 95% CI: 0.99–1.00, *p* < 0.001), and LBD pathology (PRR: 0.99, 95% CI 0.99–1.00, *p* = 0.02). In contrast, a significant positive interaction was observed for CAA (PRR: 1.00, 95% CI 0.99–1.00, *p* = 0.03, Fig. 2, Supplemental Table 3) and hemorrhages/microbleeds (PRR: 1.03, 95% CI 1.01–1.05, *p* = 0.005), indicating that the association between *APOE ε4* and microbleeds strengthens at older ages. Findings for other pathologies, including TDP-43, hippocampal sclerosis, arteriolosclerosis, atherosclerosis of the circle of Willis, CAA, gross infarcts/lacunes, and microinfarcts, did not show significant *APOE* ε4-by-age interactions, though age remained a significant predictor of increased burden for several of these pathologies.

To further examine the significant interactions between *APOE* ε4 and age at death, we conducted stratified analyses for each pathology outcome based on *APOE* ε4 carrier status (Supplemental Table 4). Among *APOE* ε4 non-carriers, increasing age at death was significantly associated with a higher prevalence of neuritic plaques (PRR = 1.01, 95% CI 1.01–1.01, *p* < 0.001), Braak staging (PRR = 1.01, 95% CI 1.01–1.01, *p* < 0.001), diffuse neuritic plaques (PRR = 1.01, 95% CI 1.01–1.01, *p* < 0.001), and CAA (PRR = 1.01, 95% CI 1.00–1.01, *p* < 0.001). In contrast, among *APOE* ε4 carriers, the association between age at death and neuritic plaques**,** Braak staging**,** diffuse neuritic plaques**,** and CAA remained statistically significant but was attenuated (PRRs = 1.00, *p* ≤ 0.02). Notably, the association between age at death and LBD pathology was significant only in *APOE* ε4 carriers (PRR = 0.99, 95% CI 0.99–1.00, *p* = 0.002). For hemorrhages/microbleeds**,** there was no significant association with age in non-carriers (PRR = 1.00, *p* = 0.68), but in *APOE* ε4 carriers, increasing age was associated with a higher prevalence of hemorrhages/microbleeds (PRR = 1.02, 95% CI 1.01–1.04, *p* = 0.002).

In the sensitivity analysis controlling for birth cohort, the findings remained largely consistent, suggesting that the observed relationships between *APOE* ε4, age at death, and neuropathology were not substantially confounded by birth cohort effects (Supplemental Table 5).

### Interactions between APOE ε4, age at death, and sex on neuropathological markers

**Fig. 3:**
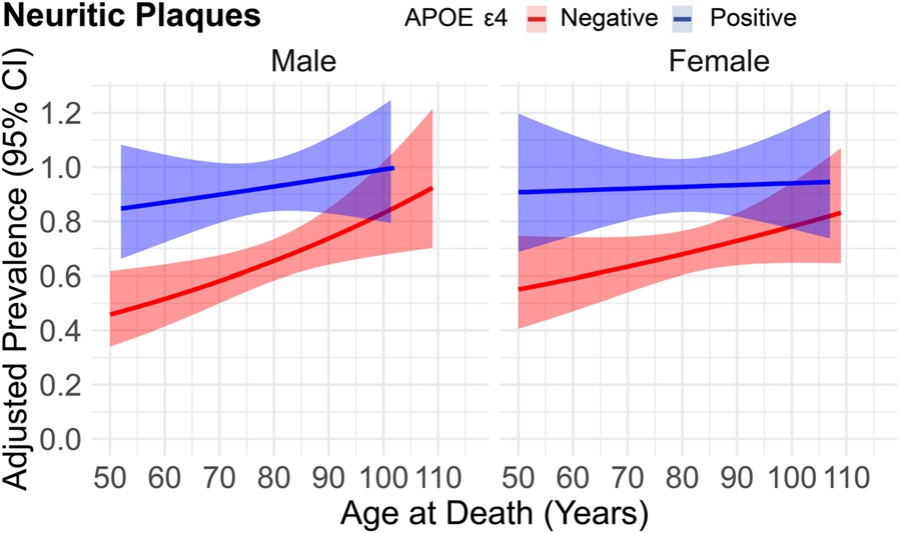
Adjusted prevalence of neuritic plaques across age groups stratified by APOE ε4 status and sex. Red lines represent individuals with *APOE* ε4 positivity, while blue lines indicate those without the APOE ε4 allele (*APOE* ε4 negative). Facets were used for male and female sex. The adjusted prevalence was derived from modified Poisson regression models adjusted for education level and included the interaction term between sex, APOE ε4 and age at death, with robust standard errors applied to account for heteroscedasticity. The line represents the mean adjusted prevalence, and the surrounding band denote 95% confidence intervals calculated from robust standard errors. Abbreviations: APOE ε4 Apolipoprotein E epsilon 4 allele; CI confidence interval

In our extended analysis evaluating the three-way interaction between *APOE* ε4, age at death, and sex, we observed key findings that further elucidate how these factors jointly influence neuropathological outcomes (Fig. 3, Supplemental Table 6). The three-way interaction between *APOE* ε4, age at death, and sex was significant for neuritic plaques (PR: 1.00, 95% CI 0.99–1.00, *p* = 0.04). No significant three-way interactions were detected for other neuropathological markers.

Among male *APOE* ε4 non-carriers, age at death was positively associated with neuritic plaque burden (PRR = 1.01, 95% CI 1.01–1.02, *p* < 0.001, Supplemental Table 7). A similar association was observed in female *APOE* ε4 non-carriers (PRR = 1.01, 95% CI 1.00–1.01, *p* = 0.002). Among male *APOE* ε4 carriers, the effect of age at death on neuritic plaques was reduced (PRR = 1.00, 95% CI 1.00–1.01, *p* = 0.002). Among female *APOE* ε4 carriers, age at death showed no significant associated with neuritic plaque burden (PRR = 1.00, 95% CI 1.00–1.00, *p* = 0.55).

## Discussion

The objective of this study was to investigate how age at death and *APOE* ε4 status interacted to influence the prevalence of autopsy-confirmed neuropathologies, including AD-related pathologies, LBD pathology, TDP-43, hippocampal sclerosis, and various vascular neuropathologies such as arteriolosclerosis, atherosclerosis of the circle of Willis, CAA, gross infarcts/lacunes, microinfarcts, and hemorrhages/microbleeds.

Our findings provide critical insights into the interplay between *APOE* ε4 status, age at death, and neuropathological outcomes in autopsy-confirmed cases. Specifically, we observed that the association between *APOE* ε4 and AD-related pathologies, including neuritic plaques, Braak staging, and diffuse plaques, as well as for LBD pathology, this association diminishes with advancing age at death. Conversely, we identified positive interactions between *APOE* ε4 and age at death for CAA and hemorrhages/microbleeds, suggesting that the influence of *APOE* ε4 on these vascular markers becomes more pronounced in older individuals.

Stratified analyses further clarified these interactions. Among *APOE* ε4 non-carriers, increasing age at death was significantly associated with higher burdens of neuritic plaques, Braak staging, diffuse plaques, and CAA. However, in *APOE* ε4 carriers, these associations remained statistically significant but were notably attenuated, supporting the hypothesis that *APOE* ε4-driven AD pathology may plateau at advanced ages. Notably, the presence of LBD pathology was significantly associated with increasing age only in *APOE* ε4 carriers, reinforcing prior findings that *APOE* ε4 may modulate synucleinopathy-related processes in a manner distinct from its role in AD pathology [45, 46]. Previous studies have shown an association between *APOE* ε4 and LBD pathology independent of AD pathology [46, 47]. Previous literature has found associations between age and hippocampal sclerosis, particularly in the context of TDP-43 pathology; however, our findings did not follow this trajectory [29]. Our findings may reflect the unique characteristics of a predominantly White and educated clinical cohort that has focused on AD primarily.

There was no significant interaction between *APOE* ε4 and age at death with arteriolosclerosis or atherosclerosis of the circle of Willis which was interesting given that *APOE* is a lipid transport protein and the similarities between the pathological progression of arteriolosclerosis and atherosclerosis. Between non-ε4 carriers and ε4 carriers, age was the strongest determinant of the majority of vascular neuropathologies which underscores the impact of aging on vascular health, which has been well-documented in previous studies [28]. The association between *APOE* ε4 and vascular neuropathologies aligns with some reports but contrasts with others, particularly those noting the role of *APOE* ε4 in macroinfarcts and arteriolosclerosis in specific populations—mostly community cohorts with smaller sample sizes [28, 48]. While hemorrhages and microbleeds were not significantly associated with age in non-carriers, *APOE* ε4 carriers exhibited a higher prevalence of these vascular lesions with increasing age. This finding is particularly relevant given that *APOE* ε4 carriers are at an elevated risk for amyloid-related imaging abnormalities following monoclonal antibody treatment, highlighting the need for careful monitoring and individualized therapeutic strategies in this population [49, 50].

In male *APOE* ε4 non-carriers, increasing age at death was associated with a greater neuritic plaque burden, a trend also observed in female non-carriers. However, among male *APOE* ε4 carriers, this effect was reduced, while in female *APOE* ε4 carriers, age was not significantly associated with neuritic plaque burden. These results suggest that female *APOE* ε4 carriers may experience an earlier saturation of amyloid pathology, which aligns with prior reports of heightened AD risk in females who carry the *APOE* ε4 allele [51, 52].

Collectively, our findings challenge the notion that *APOE* ε4 carriers remain on a linear trajectory of increasing AD pathology with advancing age. Instead, they suggest a more nuanced model in which *APOE* ε4 carriers exhibit an earlier onset and accumulation of AD pathology, though these associations weaken in the oldest individuals. In contrast, vascular outcomes such as CAA and microbleeds become more strongly associated with *APOE* ε4 status in later life, highlighting the evolving role of *APOE* ε4 across the aging continuum.

### Limitations

This study has several limitations. First, the NACC dataset is heavily skewed toward patients with dementia, introducing potential selection bias, particularly for AD-related pathologies [20, 53]. This limits the generalizability of our findings to broader populations, including individuals without AD dementia. Additionally, the cohort is predominantly White, limiting the generalizability of results to more racially and ethnically diverse groups. Another aspect of this limitation aligns with the observed attenuation of effects in older age groups may partially reflect survivorship bias, whereby individuals with more severe neuropathology may have an earlier onset of death and were underrepresented in the NACC neuropathology dataset. This is specifically for the cases and associations regarding AD pathology given that the attenuation between age and LBD has been documented previously and is well characterized [20].

Second, our study examined the prevalence of neuropathologies without stratifying by clinical diagnoses. While this approach minimizes diagnostic discrepancies, it may overlook important clinical correlations and symptom progression. We also did not account for familial AD, which could influence neuropathological associations in younger participants; however, autosomal dominant AD was rare in this cohort.

Third, although we included assessments of common neuropathologies, we did not exclude rare neurodegenerative conditions such as prion diseases, which may have impacted our conclusions. Additionally, our cross-sectional design prevents direct assessment of disease progression. Longitudinal studies are needed to quantify neuropathology in vivo and establish temporal relationships with genetic and environmental factors. Fourth, the inclusion of cognitively normal individuals at advanced ages may introduce bias, as some of these individuals could be classified as SuperAgers. SuperAgers are those over the age of 80 who maintain cognitive function comparable to individuals decades younger [54]. If these individuals exhibit resilience to neuropathological accumulation, their inclusion could skew analytical approaches and complicate the interpretation of age-related pathology trends. However, some literature contradicts this hypothesis, suggesting that normative cognitive performance in SuperAgers is not due to a lack of AD pathology accumulation, but rather to other neuroprotective mechanisms that compensate and confer resistance to cognitive decline [55].

Finally, we did not examine regional variation in neuropathological burden, such as LBD pathology, despite evidence that *APOE* ε4 is linked to LBD progression [47]. Future studies should address these limitations to improve the generalizability and clinical relevance of neuropathological research.

### Strengths

Despite its limitations, this study has several notable strengths. First, we employed modified Poisson regression models, which provide more accurate estimates of associations by reducing the potential inflation of odds ratios that can occur with logistic regression [22]. This methodological approach strengthens the reliability of our findings, particularly in studies with binary outcomes [22].

Second, our study utilized data from 5,843 individuals in the NACC database, offering substantial statistical power to detect associations, including those for less common neuropathologies, such as TDP-43. This large and well-characterized dataset allows for a comprehensive examination of neuropathological outcomes across a range of conditions, including AD-related pathologies, LBD, TDP-43, hippocampal sclerosis, and multiple vascular pathologies.

Third, by analyzing the interaction between age at death and *APOE* ε4 genotype, our study provides nuanced insights into how these factors differentially influence neuropathological burden. This distinction underscores the importance of considering both genetic and age-related factors when evaluating neurodegenerative disease risk. Overall, the combination of a robust statistical framework, a large and diverse neuropathological sample, and a targeted focus on *APOE* ε4 and age at death interactions strengthens the validity and clinical relevance of our study’s findings.

## Conclusions and future directions

Our findings demonstrate that *APOE* ε4 and age interact in complex ways to influence neuropathological outcomes, with distinct effects across AD-related and vascular pathologies. Our findings reinforce the necessity of age-stratified approaches in AD and cerebrovascular research, particularly in biomarker studies and clinical trials targeting *APOE* ε4 carriers. Specifically, our results suggest that interventions targeting *APOE* ε4-associated AD pathology may need to be implemented earlier in life, whereas vascular-targeted strategies may be more relevant at later ages. Future research should investigate the mechanisms underlying these age-dependent shifts, including potential interactions with genetic, metabolic, and inflammatory pathways that modulate neuropathological progression.

In summary, our study underscores the dynamic nature of *APOE* ε4 effects across the lifespan, with implications for both AD and vascular pathogenesis. The weakening of *APOE* ε4 associations with AD neuropathology at older ages, coupled with the increasing impact of *APOE* ε4 on vascular pathology, suggests that disease risk among *APOE* ε4 carriers is neither uniform nor static. These findings highlight the importance of personalized risk assessment and intervention strategies tailored to different stages of aging.

Our methodological approach further emphasizes the value of examining interactive effects rather than assuming static risk contributions. Future research should expand these investigations to diverse and underrepresented racial/ethnic groups, as such analyses are critical to understanding how *APOE* ε4 and age interact across populations with differing genetic backgrounds and environmental exposures. Additionally, longitudinal studies incorporating clinical outcomes will be essential to determine how these neuropathological interactions contribute to cognitive decline and functional impairment, ultimately informing precision medicine strategies for AD and related dementias.

## Supplementary Information

Additional file 1.

Additional file 2.

Additional file 3.

Additional file 4.

Additional file 5.

Additional file 6.

## Data Availability

All data used in the current analyses is available for download from the NACC (https://www.alz.washington.edu/).

## Abbreviations

*APOE*: Apolipoprotein E
LOAD: Late-onset Alzheimer's disease
NACC: National Alzheimer’s Coordinating Center
ADRC: Alzheimer’s Disease Research Centers
ADNC: Alzheimer’s disease neuropathology change
CERAD: Consortium to Establish a Registry for Alzheimer’s Disease
ABC: National Institutes of Aging-Alzheimer’s Association Alzheimer’s disease neuropathologic change ABC score
LBD: Lewy body disease
TDP-43: TAR DNA-binding protein 43
CAA: Cerebral amyloid angiopathy
UDS: Uniform Data Set
MCI: Mild cognitive impairment
PR: Prevalence ratios
CI: 95% Confidence intervals
ARR: Adjusted rate ratios

## References

[1] European Alzheimer’s Disease Initiative (EADI) et al (2013) Meta-analysis of 74,046 individuals identifies 11 new susceptibility loci for Alzheimer’s disease. Nat Genet 45(12):1452–1458. 10.1038/ng.2802 24162737 10.1038/ng.2802 PMC3896259

[2] Corder EH et al (1994) Protective effect of apolipoprotein E type 2 allele for late onset Alzheimer disease. Nat Genet 7(2):180–184. 10.1038/ng0694-180 7920638 10.1038/ng0694-180

[3] Farrer LA et al (1997) Effects of age, sex, and ethnicity on the association between apolipoprotein E genotype and Alzheimer disease. A meta-analysis. APOE and Alzheimer disease meta analysis consortium. JAMA 278(16):1349–1356 9343467

[4] Sando SB et al (2008) APOE epsilon 4 lowers age at onset and is a high risk factor for Alzheimer’s disease; a case control study from central Norway. BMC Neurol 8:9. 10.1186/1471-2377-8-9 18416843 10.1186/1471-2377-8-9 PMC2375917

[5] Corder EH et al (1993) Gene dose of apolipoprotein E type 4 Allele and the risk of alzheimer’s disease in late onset families. Science 261(5123):921–923. 10.1126/science.8346443 8346443 10.1126/science.8346443

[6] Mahley RW, Rall SC (2000) Apolipoprotein E: far more than a lipid transport protein. Annu Rev Genom Hum Genet 1(1):507–537. 10.1146/annurev.genom.1.1.507 10.1146/annurev.genom.1.1.507 11701639

[7] Tudorache IF, Trusca VG, Gafencu AV (2017) Apolipoprotein E - a multifunctional protein with implications in various pathologies as a result of its structural features. Comput Struct Biotechnol J 15:359–365. 10.1016/j.csbj.2017.05.003 28660014 10.1016/j.csbj.2017.05.003 PMC5476973

[8] Bras J et al (2014) Genetic analysis implicates APOE, SNCA and suggests lysosomal dysfunction in the etiology of dementia with Lewy bodies. Hum Mol Genet 23(23):6139–6146. 10.1093/hmg/ddu334 24973356 10.1093/hmg/ddu334 PMC4222357

[9] Guerreiro R et al (2018) Investigating the genetic architecture of dementia with Lewy bodies: a two-stage genome-wide association study. Lancet Neurol 17(1):64–74. 10.1016/S1474-4422(17)30400-3 29263008 10.1016/S1474-4422(17)30400-3 PMC5805394

[10] Huang X, Chen P, Kaufer DI, Tröster AI, Poole C (2006) Apolipoprotein E and dementia in Parkinson disease: a meta-analysis. Arch Neurol 63(2):189–193. 10.1001/archneur.63.2.189 16476806 10.1001/archneur.63.2.189

[11] Irwin DJ et al (2012) Neuropathologic substrates of Parkinson disease dementia. Ann Neurol 72(4):587–598. 10.1002/ana.23659 23037886 10.1002/ana.23659 PMC3484250

[12] Tropea TF et al (2018) APOE, thought disorder, and SPARE-AD predict cognitive decline in established Parkinson’s disease. Mov Disord Off J Mov Disord Soc 33(2):289–297. 10.1002/mds.27204 10.1002/mds.27204 PMC5809205 29168904

[13] Tsuang D et al (2013) APOE ε4 increases risk for dementia in pure synucleinopathies. JAMA Neurol 70(2):223–228. 10.1001/jamaneurol.2013.600 23407718 10.1001/jamaneurol.2013.600 PMC3580799

[14] Josephs KA et al (2014) TDP-43 is a key player in the clinical features associated with Alzheimer’s disease. Acta Neuropathol (Berl) 127(6):811–824. 10.1007/s00401-014-1269-z 24659241 10.1007/s00401-014-1269-z PMC4172544

[15] Wennberg AM et al (2018) Association of apolipoprotein E ε4 With transactive response DNA-binding protein 43. JAMA Neurol 75(11):1347–1354. 10.1001/jamaneurol.2018.3139 30422173 10.1001/jamaneurol.2018.3139 PMC6248121

[16] Yang H-S et al (2018) Evaluation of TDP-43 proteinopathy and hippocampal sclerosis in relation to APOE ε4 haplotype status: a community-based cohort study. Lancet Neurol 17(9):773–781. 10.1016/S1474-4422(18)30251-5 30093249 10.1016/S1474-4422(18)30251-5 PMC6154505

[17] Zannis VI et al (1982) Proposed nomenclature of apoE isoproteins, apoE genotypes, and phenotypes. J Lipid Res 23(6):911–914 7130859

[18] Qian J, Betensky RA, Hyman BT, Serrano-Pozo A (2021) Association of *APOE* genotype with heterogeneity of cognitive decline rate in Alzheimer disease. Neurology. 10.1212/WNL.0000000000011883 33771840 10.1212/WNL.0000000000011883 PMC8166439

[19] Besser LM et al (2018) The revised national alzheimer’s coordinating center’s neuropathology form—available data and new analyses. J Neuropathol Exp Neurol 77(8):717–726. 10.1093/jnen/nly049 29945202 10.1093/jnen/nly049 PMC6044344

[20] Beach TG, Malek-Ahmadi M (2021) Alzheimer’s disease neuropathological comorbidities are common in the younger-old. J Alzheimers Dis 79(1):389–400. 10.3233/JAD-201213 33285640 10.3233/JAD-201213 PMC8034496

[21] McNutt L-A (2003) Estimating the relative risk in cohort studies and clinical trials of common outcomes. Am J Epidemiol 157(10):940–943. 10.1093/aje/kwg074 12746247 10.1093/aje/kwg074

[22] Zou G (2004) A modified poisson regression approach to prospective studies with binary data. Am J Epidemiol 159(7):702–706. 10.1093/aje/kwh090 15033648 10.1093/aje/kwh090

[23] Tiraboschi P, Hansen LA, Masliah E, Alford M, Thal LJ, Corey-Bloom J (2004) Impact of *APOE* genotype on neuropathologic and neurochemical markers of Alzheimer disease. Neurology 62(11):1977–1983. 10.1212/01.WNL.0000128091.92139.0F 15184600 10.1212/01.wnl.0000128091.92139.0f

[24] Benjamin R et al (1994) Protective effect of apoE &isin;2 in Alzheimer’s disease. The Lancet 344(8920):473–474. 10.1016/S0140-6736(94)91804-X 10.1016/s0140-6736(94)91804-x 7914580

[25] Toledo JB et al (2013) Contribution of cerebrovascular disease in autopsy confirmed neurodegenerative disease cases in the National Alzheimer’s Coordinating Centre. Brain 136(9):2697–2706. 10.1093/brain/awt188 23842566 10.1093/brain/awt188 PMC3858112

[26] Hyman BT et al (2012) National Institute on Aging–Alzheimer’s Association guidelines for the neuropathologic assessment of Alzheimer’s disease. Alzheimers Dement 8(1):1–13. 10.1016/j.jalz.2011.10.007 22265587 10.1016/j.jalz.2011.10.007 PMC3266529

[27] Montine TJ et al (2012) National Institute on Aging–Alzheimer’s Association guidelines for the neuropathologic assessment of Alzheimer’s disease: a practical approach. Acta Neuropathol (Berl) 123(1):1–11. 10.1007/s00401-011-0910-3 22101365 10.1007/s00401-011-0910-3 PMC3268003

[28] Lamar M et al (2019) *APOE* genotypes as a risk factor for age-dependent accumulation of cerebrovascular disease in older adults. Alzheimers Dement 15(2):258–266. 10.1016/j.jalz.2018.08.007 30321502 10.1016/j.jalz.2018.08.007 PMC6368888

[29] Maldonado-Díaz C et al (2024) Disentangling and quantifying the relative cognitive impact of concurrent mixed neurodegenerative pathologies. Acta Neuropathol (Berl) 147(1):58. 10.1007/s00401-024-02716-y 38520489 10.1007/s00401-024-02716-y PMC10960766

[30] Wilson RS et al (2019) Postmortem neurodegenerative markers and trajectories of decline in cognitive systems. Neurology 92(8):e831–e840. 10.1212/WNL.0000000000006949 30674595 10.1212/WNL.0000000000006949 PMC6396970

[31] Frank B et al (2022) Trajectories of cognitive decline in brain donors with autopsy-confirmed Alzheimer disease and cerebrovascular disease. Neurology 98(24):e2454–e2464. 10.1212/WNL.0000000000200304 35444054 10.1212/WNL.0000000000200304 PMC9231841

[32] Phongpreecha T et al (2023) Prediction of neuropathologic lesions from clinical data. Alzheimers Dement. 10.1002/alz.12921 36681388 10.1002/alz.12921 PMC10359434

[33] Braak H, Alafuzoff I, Arzberger T, Kretzschmar H, Del Tredici K (2006) Staging of Alzheimer disease-associated neurofibrillary pathology using paraffin sections and immunocytochemistry. Acta Neuropathol (Berl) 112(4):389–404. 10.1007/s00401-006-0127-z 16906426 10.1007/s00401-006-0127-z PMC3906709

[34] Ryman SG et al (2021) Cognition at each stage of lewy body disease with co-occurring alzheimer’s disease pathology1. J Alzheimers Dis 80(3):1243–1256. 10.3233/JAD-201187 33646154 10.3233/JAD-201187 PMC8150665

[35] Amador-Ortiz C et al (2007) TDP-43 immunoreactivity in hippocampal sclerosis and Alzheimer’s disease. Ann Neurol 61(5):435–445. 10.1002/ana.21154 17469117 10.1002/ana.21154 PMC2677204

[36] Amador-Ortiz C, Dickson DW (2008) Neuropathology of hippocampal sclerosis. Handb Clin Neurol 89:569–572. 10.1016/S0072-9752(07)01253-5 18631779 10.1016/S0072-9752(07)01253-5

[37] Barnes LL et al (2015) Mixed pathology is more likely in black than white decedents with Alzheimer dementia. Neurology 85(6):528–534. 10.1212/WNL.0000000000001834 26180136 10.1212/WNL.0000000000001834 PMC4540250

[38] Lammie GA, Brannan F, Slattery J, Warlow C (1997) Nonhypertensive cerebral small-vessel disease: an autopsy study. Stroke 28(11):2222–2229. 10.1161/01.STR.28.11.2222 9368569 10.1161/01.str.28.11.2222

[39] Viswanathan A, Greenberg SM (2011) Cerebral amyloid angiopathy in the elderly. Ann Neurol 70(6):871–880. 10.1002/ana.22516 22190361 10.1002/ana.22516 PMC4004372

[40] Brenowitz WD et al (2017) Mixed neuropathologies and associations with domain-specific cognitive decline. Neurology 89(17):1773–1781. 10.1212/WNL.0000000000004567 28939667 10.1212/WNL.0000000000004567 PMC5664309

[41] Wardlaw JM (2008) What is a lacune? Stroke 39(11):2921–2922. 10.1161/STROKEAHA.108.523795 18703799 10.1161/STROKEAHA.108.523795

[42] Arvanitakis Z, Leurgans SE, Barnes LL, Bennett DA, Schneider JA (2011) Microinfarct pathology, dementia, and cognitive systems. Stroke 42(3):722–727. 10.1161/STROKEAHA.110.595082 21212395 10.1161/STROKEAHA.110.595082 PMC3042494

[43] Vinters HV et al (2000) Neuropathologic substrates of ischemic vascular dementia. J Neuropathol Exp Neurol 59(11):931–945. 10.1093/jnen/59.11.931 11089571 10.1093/jnen/59.11.931

[44] Thorpe RJ et al (2020) The association between depressive symptoms and accumulation of stress among black men in the health and retirement study. Innov Aging 4(5):047. 10.1093/geroni/igaa047 10.1093/geroni/igaa047 PMC7737789 33354627

[45] Davis AA et al (2020) *APOE* genotype regulates pathology and disease progression in synucleinopathy. Sci Transl Med 12(529):eaay3069. 10.1126/scitranslmed.aay3069 32024799 10.1126/scitranslmed.aay3069 PMC7289511

[46] Zhao N et al (2020) APOE4 exacerbates α-synuclein pathology and related toxicity independent of amyloid. Sci Transl Med 529:eaay1809. 10.1126/scitranslmed.aay1809 10.1126/scitranslmed.aay1809 PMC8309690 32024798

[47] Dickson DW et al (2018) *APOE* ε4 is associated with severity of Lewy body pathology independent of Alzheimer pathology. Neurology. 10.1212/WNL.0000000000006212 30143564 10.1212/WNL.0000000000006212 PMC6161556

[48] Goldberg TE, Huey ED, Devanand DP (2021) Associations of APOE e2 genotype with cerebrovascular pathology: a postmortem study of 1275 brains. J Neurol Neurosurg Psychiatry 92(1):7–11. 10.1136/jnnp-2020-323746 10.1136/jnnp-2020-323746 PMC11299059 33148816

[49] Van Dyck CH et al (2023) Lecanemab in early Alzheimer’s disease. N Engl J Med 388(1):9–21. 10.1056/NEJMoa2212948 36449413 10.1056/NEJMoa2212948

[50] Cogswell PM et al (2022) Amyloid-related imaging abnormalities with emerging alzheimer disease therapeutics: detection and reporting recommendations for clinical practice. Am J Neuroradiol 43(9):E19–E35. 10.3174/ajnr.A7586 35953274 10.3174/ajnr.A7586 PMC9451628

[51] Nemes S et al (2023) Sex and APOE ε4 carrier effects on atrophy, amyloid PET, and tau PET burden in early-onset Alzheimer’s disease. Alzheimers Dement. 10.1002/alz.13403 37496307 10.1002/alz.13403 PMC10811272

[52] Polsinelli AJ, Lane KA, Manchella MK, Logan PE, Gao S, Apostolova LG (2023) *APOE* ε4 is associated with earlier symptom onset in LOAD but later symptom onset in EOAD. Alzheimers Dement 19(5):2212–2217. 10.1002/alz.12955 36722399 10.1002/alz.12955 PMC10182241

[53] Mehta KM et al (2008) Race/ethnic DIFFERENCES in AD survival in US Alzheimer’s disease centers. Neurology 70(14):1163–1170. 10.1212/01.wnl.0000285287.99923.3c 18003939 10.1212/01.wnl.0000285287.99923.3c PMC2830859

[54] Harrison TM, Weintraub S, Mesulam M-M, Rogalski E (2012) Superior memory and higher cortical volumes in unusually successful cognitive aging. J Int Neuropsychol Soc 18(6):1081–1085. 10.1017/S1355617712000847 23158231 10.1017/S1355617712000847 PMC3547607

[55] Dominguez EN, Corrada MM, Kawas CH, Stark CEL (2024) Resilience to AD pathology in top cognitive performers. Front Aging Neurosci 16:1428695. 10.3389/fnagi.2024.1428695 39055052 10.3389/fnagi.2024.1428695 PMC11270559

