# Supplementary material for: Age-dependent interactions of APOE isoform 4 and Alzheimer’s disease neuropathology: findings from the NACC
Source: Acta Neuropathol Commun. 2025 May 17;13:102. doi: 10.1186/s40478-025-02012-0 (PMC12085078; doi:10.1186/s40478-025-02012-0)
Supplement: Supplementary file 1 — Additional file 1. [file 40478_2025_2012_MOESM1_ESM.docx]

| Supplemental Table 1: The Percentages of Pre-Mortem Clinical Etiological Diagnoses Across Decadal Groups | | | | | | |
| --- | --- | --- | --- | --- | --- | --- |
| Clinical Etiology | 50 – 59 Years (N=202) | 60 – 69 Years (N=842) | 70 – 79 Years (N=1399) | 80 – 89 Years (N=2043) | 90 + Years (N=1357) | Overall (N=5843) |
| Alzheimer’s Disease (AD), n (%) | 85 (42.1) | 381 (45.2) | 759 (54.3) | 1374 (67.3) | 860 (63.4) | 3459 (59.2) |
| Lewy Body Disease (LBD), n (%) | 4 (2.0) | 54 (6.4) | 155 (11.1) | 151 (7.4) | 37 (2.7) | 401 (6.9) |
| Progressive Supranuclear Palsy (PSP), n (%) | 8 (4.0) | 29 (3.4) | 39 (2.8) | 19 (0.9) | 0 (0) | 95 (1.6) |
| Corticobasal Degeneration (CBD), n (%) | 3 (1.5) | 48 (5.7) | 39 (2.8) | 12 (0.6) | 0 (0) | 102 (1.7) |
| FTLD with motor neuron disease (e.g., ALS), n (%) | 3 (1.5) | 8 (1.0) | 5 (0.4) | 0 (0) | 0 (0) | 16 (0.3) |
| FTLD, other, n (%) | 69 (34.2) | 252 (29.9) | 242 (17.3) | 67 (3.3) | 8 (0.6) | 638 (10.9) |
| Vascular Brain Injury or Vascular Dementia including Stroke, n (%) | 0 (0) | 4 (0.5) | 9 (0.6) | 66 (3.2) | 73 (5.4) | 152 (2.6) |
| Down Syndrome, n (%) | 1 (0.5) | 0 (0) | 0 (0) | 0 (0) | 0 (0) | 1 (0.0) |
| Huntington’s Disease, n (%) | 0 (0) | 0 (0) | 1 (0.1) | 0 (0) | 0 (0) | 1 (0.0) |
| Prion Disease, n (%) | 4 (2.0) | 3 (0.4) | 1 (0.1) | 1 (0.0) | 0 (0) | 9 (0.2) |
| Traumatic Brain Injury (TBI), n (%) | 0 (0) | 0 (0) | 2 (0.1) | 2 (0.1) | 4 (0.3) | 8 (0.1) |
| Normal Pressure Hydrocephalus, n (%) | 0 (0) | 0 (0) | 3 (0.2) | 1 (0.0) | 0 (0) | 4 (0.1) |
| CNS Neoplasm, n (%) | 0 (0) | 0 (0) | 1 (0.1) | 0 (0) | 0 (0) | 1 (0.0) |
| Other Neurologic, Genetic, or Infectious Condition, n (%) | 0 (0) | 5 (0.6) | 0 (0) | 9 (0.4) | 7 (0.5) | 21 (0.4) |
| Depression, n (%) | 0 (0) | 3 (0.4) | 1 (0.1) | 8 (0.4) | 6 (0.4) | 18 (0.3) |
| Bipolar Disorder, n (%) | 0 (0) | 1 (0.1) | 0 (0) | 0 (0) | 0 (0) | 1 (0.0) |
| Schizophrenia, n (%) | 0 (0) | 0 (0) | 0 (0) | 0 (0) | 1 (0.1) | 1 (0.0) |
| Other Psychiatric Disease, n (%) | 0 (0) | 0 (0) | 0 (0) | 1 (0.0) | 0 (0) | 1 (0.0) |
| Alcohol Abuse, n (%) | 0 (0) | 2 (0.2) | 0 (0) | 3 (0.1) | 1 (0.1) | 6 (0.1) |
| Cognitive Impairment, Systemic Illness or Medical Illness, n (%) | 0 (0) | 2 (0.2) | 3 (0.2) | 19 (0.9) | 14 (1.0) | 38 (0.7) |
| Cognitive Impairment, Medications, n (%) | 0 (0) | 0 (0) | 1 (0.1) | 4 (0.2) | 1 (0.1) | 6 (0.1) |
| Cognitive Impairment, Other Reasons, n (%) | 10 (5.0) | 17 (2.0) | 20 (1.4) | 20 (1.0) | 11 (0.8) | 78 (1.3) |
| Not Cognitively Impaired, n (%) | 9 (4.5) | 21 (2.5) | 100 (7.1) | 255 (12.5) | 295 (21.7) | 680 (11.6) |
| Missing/Unknown, n (%) | 6 (3.0) | 12 (1.4) | 18 (1.3) | 31 (1.5) | 39 (2.9) | 106 (1.8) |
| This table presents the percentages of various pre-mortem clinical etiological diagnoses stratified by age groups (50–59 years, 60–69 years, 70–79 years, 80–89 years, and 90+ years) along with the overall percentages. Each row represents a specific diagnosis, with the corresponding counts (N) and percentages (%) for each age group and the overall population (N=5843).  Abbreviations: AD Alzheimer’s Disease; LBD Lewy Body Disease; PSP Progressive Supranuclear Palsy; CBD Corticobasal Degeneration; FTLD Frontotemporal Lobar Degeneration; ALS Amyotrophic Lateral Sclerosis; TBI Traumatic Brain Injury; CNS Central Nervous System; CAA Cerebral Amyloid Angiopathy; VBI Vascular Brain Injury; NPH Normal Pressure Hydrocephalus; MCI Mild Cognitive Impairment. | | | | | | |
