# Supplementary material for: Age-dependent interactions of APOE isoform 4 and Alzheimer’s disease neuropathology: findings from the NACC
Source: Acta Neuropathol Commun. 2025 May 17;13:102. doi: 10.1186/s40478-025-02012-0 (PMC12085078; doi:10.1186/s40478-025-02012-0)
Supplement: Supplementary file 2 — Additional file 2. [file 40478_2025_2012_MOESM2_ESM.docx]

| Supplemental Table 2: The Distribution of Dominantly Inherited Alzheimer’s Disease Mutation and CADASIL Across Decadal Groups | | | | | | |
| --- | --- | --- | --- | --- | --- | --- |
|  | 50 – 59 Years (N=202) | 60 – 69 Years (N=842) | 70 – 79 Years (N=1399) | 80 – 89 Years (N=2043) | 90 + Years (N=1357) | Overall (N=5843) |
| Dominantly  Inherited AD Mutation, n (%) | 13 (6.4) | 10 (1.2) | 3 (0.2) | 2 (0.1) | 2 (0.1) | 30 (0.5) |
| CADASIL, n (%) | 0 (0) | 0 (0) | 0 (0) | 1 (0.0) | 1 (0.1) | 2 (0.0) |
| Abbreviations: AD (Alzheimer’s Disease); Cerebral Autosomal Dominant Arteriopathy with Subcortical Infarcts and Leukoencephalopathy (CADASIL) | | | | | | |
