# Supplementary material for: Age-dependent interactions of APOE isoform 4 and Alzheimer’s disease neuropathology: findings from the NACC
Source: Acta Neuropathol Commun. 2025 May 17;13:102. doi: 10.1186/s40478-025-02012-0 (PMC12085078; doi:10.1186/s40478-025-02012-0)
Supplement: Supplementary file 3 — Additional file 3. [file 40478_2025_2012_MOESM3_ESM.docx]

| Supplemental Table 3: Prevalence Ratios for Neuropathologies by APOE ε4 Status and Age at Death and their Interactions | | | |
| --- | --- | --- | --- |
| Outcome and Sample Size | *APOE ε4* PRR (95% CI), p-value | Age at Death  PRR (95% CI), p-value | *APOE ε4* x Age at Death  PRR (95% CI), p-value |
| Neuritic plaques, N=5763 | **2.54 (2.02 - 3.20), p<0.001** | **1.01 (1.01 - 1.01), p<0.001** | **0.99 (0.99 - 1.00), p<0.001** |
| Braak staging, N=5678 | **1.77 (1.55 - 2.01), p<0.001** | **1.01 (1.01 - 1.01), p<0.001** | **0.99 (0.99 - 1.00), p<0.001** |
| Diffuse plaques, N=5298 | **1.86 (1.55 - 2.24), p<0.001** | **1.01 (1.01 - 1.01), p<0.001** | **0.99 (0.99 - 1.00), p<0.001** |
| LBD pathology, N=5523 | **2.70 (1.57 - 4.64), p<0.001** | 1.00 (1.00 - 1.01), p=0.73 | **0.99 (0.99 - 1.00), p=0.02** |
| TDP-43, N=2049 | 0.70 (0.34 - 1.45), p=0.34 | **1.01 (1.00 - 1.01), p=0.04** | 1.01 (1.00 - 1.02), p=0.10 |
| Hippocampal sclerosis, N=3212 | 0.55 (0.15 - 2.00), p=0.36 | **1.01 (1.00 - 1.03), p=0.01** | 1.01 (1.00 - 1.03), p=0.18 |
| Arteriolosclerosis, N=5195 | 0.91 (0.73 - 1.13), p=0.38 | **1.00 (1.00 - 1.01), p<0.001** | 1.00 (1.00 - 1.00), p=0.22 |
| Atherosclerosis of the circle of Willis, N=5703 | 0.93 (0.71 - 1.21), p=0.58 | **1.01 (1.01 - 1.02), p<0.001** | 1.00 (1.00 - 1.00), p=0.40 |
| Cerebral Amyloid Angiopathy, N=5672 | **2.38 (1.71 - 3.31), p<0.001** | **1.01 (1.00 - 1.01), p<0.001** | **1.00 (0.99 - 1.00), p=0.03** |
| Infarcts/lacunes, N=5732 | 0.73 (0.29 - 1.85), p=0.51 | **1.04 (1.03 - 1.05), p<0.001** | 1.00 (0.99 - 1.02), p=0.42 |
| Microinfarcts, N=5734 | 0.94 (0.38 - 2.29), p=0.89 | **1.04 (1.03 - 1.05), p<0.001** | 1.00 (0.99 - 1.01), p=0.91 |
| Hemorrhages/ microbleeds, N=5622 | **0.12 (0.02 - 0.53), p=0.006** | 1.00 (0.99 - 1.01), p=0.64 | **1.03 (1.01 - 1.05), p=0.005** |
| Model: pathology outcome ~ centered education + sex + APOE ε4 * age at death (years). The N represents the total sample size used in the regression model considering that some pathologies were missing in participants.  Data are presented as prevalence rate ratios and 95% confidence intervals.  Bold indicates significance.  Abbreviations: *APOE* apolipoprotein E epsilon 4; PRR prevalence rate ratio; CI confidence interval; LBD Lewy Body  Disease Pathology; TDP-43 Transactive response DNA binding protein 43 | | | |
