# Supplementary material for: Age-dependent interactions of APOE isoform 4 and Alzheimer’s disease neuropathology: findings from the NACC
Source: Acta Neuropathol Commun. 2025 May 17;13:102. doi: 10.1186/s40478-025-02012-0 (PMC12085078; doi:10.1186/s40478-025-02012-0)
Supplement: Supplementary file 4 — Additional file 4. [file 40478_2025_2012_MOESM4_ESM.docx]

| Supplemental Table 4: Stratified Analyses from Significant Interactions between APOE ε4 and Age at Death | | |
| --- | --- | --- |
| Outcome | Stratification | Age at Death  PRR (95% CI), p-value |
| Neuritic Plaques | *APOE ε4 Non-Carrier* | 1.01 (1.01 - 1.01), p<0.001 |
|  | *APOE ε4 Carrier* | 1.00 (1.00 - 1.00), p=0.006 |
| Braak Staging | *APOE ε4 Non-Carrier* | 1.01 (1.01 - 1.01), p<0.001 |
|  | *APOE ε4 Carrier* | 1.00 (1.00 - 1.00), p<0.001 |
| Diffuse Neuritic Plaques | *APOE ε4 Non-Carrier* | 1.01 (1.01 - 1.01), p<0.001 |
|  | *APOE ε4 Carrier* | 1.00 (1.00 - 1.00), p<0.001 |
| LBD Pathology | *APOE ε4 Non-Carrier* | 1.00 (1.00 - 1.01), p=0.50 |
|  | *APOE ε4 Carrier* | 0.99 (0.99 - 1.00), p=0.002 |
| Cerebral Amyloid Angiopathy | *APOE ε4 Non-Carrier* | 1.01 (1.00 - 1.01), p<0.001 |
|  | *APOE ε4 Carrier* | 1.00 (1.00 - 1.00), p=0.02 |
| Hemorrhages/ microbleeds | *APOE ε4 Non-Carrier* | 1.00 (0.99 - 1.01), p=0.68 |
|  | *APOE ε4 Carrier* | 1.02 (1.01 - 1.04), p=0.002 |
| Model: pathology outcome ~ centered education + sex + age at death (years) stratified by *APOE ε4* carrier status.  Data are presented as prevalence rate ratios and 95% confidence intervals.  Abbreviations: *APOE* apolipoprotein E epsilon 4; PRR prevalence rate ratio; CI confidence interval; LBD Lewy Body Disease Pathology | | |
