# Supplementary material for: Age-dependent interactions of APOE isoform 4 and Alzheimer’s disease neuropathology: findings from the NACC
Source: Acta Neuropathol Commun. 2025 May 17;13:102. doi: 10.1186/s40478-025-02012-0 (PMC12085078; doi:10.1186/s40478-025-02012-0)
Supplement: Supplementary file 5 — Additional file 5. [file 40478_2025_2012_MOESM5_ESM.docx]

| Supplementary Table 6: Interaction Associations of Sex, *APOE ε4*, and Age at Death on Prevalence Rate of Neuropathology | | | | | | |
| --- | --- | --- | --- | --- | --- | --- |
| Outcome and Sample Size | *APOE ε4* PRR (95% CI), p-value | Age at Death  PRR (95% CI), p-value | Sex (Male vs. Female) PRR (95% CI), p-value | *APOE ε4 ** Age at Death  PRR (95% CI), p-value | Sex * Age at Death  PRR (95% CI), p-value | Sex * *APOE ε4 ** Age at Death  PRR (95% CI), p-value |
| Neuritic plaques, N=5763 | **2.81 (2.02 - 3.91), p<0.001** | **1.01 (1.01 - 1.02), p<0.001** | **1.54 (1.03 - 2.30), p=0.03** | **0.99 (0.99 - 1.00), p<0.001** | 0.80 (0.50 - 1.27), p=0.34 | **1.00 (0.99 - 1.00), p=0.04** |
| Braak staging, N=5678 | **1.69 (1.41 - 2.03), p<0.001** | **1.01 (1.01 - 1.01), p<0.001** | 0.98 (0.78 - 1.24), p=0.89 | **0.99 (0.99 - 1.00), p<0.001** | 1.13 (0.86 - 1.46), p=0.38 | 1.00 (1.00 - 1.00), p=0.96 |
| Diffuse plaques, N=5298 | **2.03 (1.56 - 2.63), p<0.001** | **1.01 (1.01 - 1.01), p<0.001** | 1.30 (0.94 - 1.81), p=0.11 | **0.99 (0.99 - 1.00), p<0.001** | 0.82 (0.56 - 1.19), p=0.30 | 1.00 (0.99 - 1.00), p=0.13 |
| LBD pathology, N=5523 | **4.04 (2.00 - 8.17), p<0.001** | 1.00 (1.00 - 1.01), p=0.15 | 1.31 (0.57 - 3.00), p=0.53 | **0.99 (0.98 - 0.99), p=0.002** | 0.51 (0.17 - 1.54), p=0.23 | 0.99 (0.98 - 1.00), p=0.15 |
| TDP-43, N=2049 | 0.28 (0.09 - 0.82), p=0.02 | 1.01 (1.00 - 1.01), p=0.20 | 0.88 (0.31 - 2.47), p=0.81 | **1.02 (1.01 - 1.03), p=0.007** | **5.97 (1.37 - 26.00), p=0.02** | 1.00 (0.99 - 1.01), p=0.78 |
| Hippocampal sclerosis, N=3212 | 0.28 (0.04 - 1.88), p=0.19 | **1.02 (1.00 - 1.03), p=0.04** | 0.87 (0.13 - 5.66), p=0.88 | 1.02 (0.99 - 1.04), p=0.18 | 5.01 (0.34 - 72.79), p=0.24 | 1.00 (0.98 - 1.02), p=0.96 |
| Arteriolosclerosis, N=5195 | 0.80 (0.59 - 1.09), p=0.15 | **1.00 (1.00 - 1.01), p=0.006** | 0.79 (0.59 - 1.07), p=0.13 | 1.00 (1.00 - 1.01), p=0.11 | 1.33 (0.86 - 2.08), p=0.20 | 1.00 (1.00 - 1.01), p=0.14 |
| Atherosclerosis of the circle of Willis, N=5703 | 0.84 (0.58 - 1.20), p=0.33 | **1.01 (1.01 - 1.02), p<0.001** | 0.96 (0.68 - 1.36), p=0.83 | 1.00 (1.00 - 1.01), p=0.28 | 1.32 (0.78 - 2.25), p=0.30 | 1.00 (1.00 - 1.00), p=0.99 |
| Cerebral Amyloid Angiopathy, N=5672 | **2.25 (1.42 - 3.57), p<0.001** | **1.01 (1.00 - 1.01), p<0.001** | 1.16 (0.66 - 2.05), p=0.61 | 1.00 (0.99 - 1.00), p=0.20 | 1.14 (0.58 - 2.22), p=0.70 | 1.00 (0.99 - 1.00), p=0.58 |
| Infarcts/lacunes, N=5732 | 0.81 (0.22 - 3.02), p=0.75 | **1.04 (1.03 - 1.05), p<0.001** | 1.46 (0.43 - 4.93), p=0.55 | 1.00 (0.99 - 1.02), p=0.74 | 0.93 (0.14 - 6.12), p=0.94 | 0.99 (0.98 - 1.01), p=0.45 |
| Microinfarcts, N=5734 | 1.16 (0.33 - 4.10), p=0.82 | **1.04 (1.03 - 1.05), p<0.001** | 0.84 (0.27 - 2.67), p=0.77 | 1.00 (0.98 - 1.01), p=0.73 | 0.75 (0.12 - 4.56), p=0.75 | 1.00 (0.99 - 1.01), p=0.88 |
| Hemorrhages/ microbleeds, N=5622 | 0.19 (0.02 - 1.84), p=0.15 | 1.00 (0.98 - 1.02), p=0.89 | 1.73 (0.27 - 11.16), p=0.57 | 1.02 (0.99 - 1.05), p=0.17 | 0.40 (0.02 - 8.69), p=0.56 | 0.99 (0.97 - 1.02), p=0.53 |
| Model: pathology outcome ~ centered education + sex * *APOE ε4* * age at death (years). The N represents the total sample size used in the regression model considering that some pathologies were missing in participants.  Data are presented as prevalence rate ratios and 95% confidence intervals.  Bold indicates significance.  Abbreviations: *APOE* apolipoprotein E epsilon 4; PRR prevalence rate ratio; CI confidence interval; LBD Lewy Body  Disease Pathology; TDP-43 Transactive response DNA binding protein 43 | | | | | | |
