# Supplementary material for: Age-dependent interactions of APOE isoform 4 and Alzheimer’s disease neuropathology: findings from the NACC
Source: Acta Neuropathol Commun. 2025 May 17;13:102. doi: 10.1186/s40478-025-02012-0 (PMC12085078; doi:10.1186/s40478-025-02012-0)
Supplement: Supplementary file 6 — Additional file 6. [file 40478_2025_2012_MOESM6_ESM.docx]

| Supplemental Table 7: Stratified Analyses for Neuritic Plaques from Significant Interactions between APOE ε4 and Age at Death and Sex | |
| --- | --- |
| Stratification | Age at Death |
| Male *APOE ε4 Non-Carriers* | 1.01 (1.01 - 1.02), p<0.001 |
| Female *APOE ε4 Non-Carriers* | 1.01 (1.00 - 1.01), p<0.002 |
| Male *APOE ε4 Carriers* | 1.00 (1.00 - 1.01), p=0.002 |
| Female *APOE ε4 Carriers* | 1.00 (1.00 - 1.00), p=0.55 |
| Model: pathology outcome ~ centered education + age at death (years), stratified by APOE ε4 carrier status and sex. The N represents the total sample size used in each stratified regression model, considering that some pathologies were missing in participants.  Data are presented as **prevalence rate ratios (PRR) with 95% confidence intervals (CI).** **Bold** indicates statistical significance.  Abbreviations: *APOE* apolipoprotein E epsilon 4; PRR prevalence rate ratio; CI confidence interval; | |
